# DEVELOPING A SENSOR-BASED MOBILE APPLICATION FOR IN-HOME FRAILTY ASSESSMENT: A QUALITATIVE STUDY

**DOI:** 10.1093/geroni/igz038.3064

**Published:** 2019-11-08

**Authors:** Marcela D Blinka, Brian Buta, Kevin Bader, Casey L Hanley, Nancy Schoenborn, Matthew McNabney, Qian-Li Xue

**Affiliations:** 1 Center on Aging and Health, Division of Geriatric Medicine and Gerontology, Johns Hopkins University School of Medicine, Baltimore, Maryland, United States; 2 The Johns Hopkins Applied Physics Laboratory, Laurel, Maryland, United States; 3 Division of Geriatric Medicine and Gerontology, Johns Hopkins University School of Medicine, Baltimore, Maryland, United States

## Abstract

Frailty is an important concept in the care of older adults, and there is great interest in incorporating user-friendly frailty assessments into research and clinical settings. In-home, sensor-based technologies may provide a more dynamic, sensitive, and accurate assessment of frailty measures. To investigate user perspectives for use of sensor-based technologies and mobile applications, we held five focus groups with community-dwelling older adults (n= 10), their informal caregivers (n=9), and medical professionals (n=8). We used qualitative inductive analysis to organize thematic content. Caregivers and care-recipients viewed the early identification of frailty as beneficial, but highlighted the need for secure data infrastructure and clear demonstration of how frailty assessment would improve care. They also expressed concerns that technology-based communication could reduce in-person interactions. Medical providers noted the utility of objective data for difficult conversations with caregivers of frail patients, but worried about resources for analyses and interpretation of sensor-based health information.

